# A gigantic marine ostracod (Crustacea: Myodocopa) trapped in mid-Cretaceous Burmese amber

**DOI:** 10.1038/s41598-018-19877-y

**Published:** 2018-01-22

**Authors:** Lida Xing, Benjamin Sames, Ryan C. McKellar, Dangpeng Xi, Ming Bai, Xiaoqiao Wan

**Affiliations:** 1 State Key Laboratory of Biogeology and Environmental Geology, China University of Geosciences, Beijing, 100083 China; 2 School of the Earth Sciences and Resources, China University of Geosciences, Beijing, 100083 China; 3 Department of Geodynamics and Sedimentology, University of Vienna, Geozentrum, Althanstrasse 14, 1090 Vienna, Austria; 4 Sam Noble Museum, 2401 Chautauqua Avenue, Norman, OK 73072 USA; 5 Royal Saskatchewan Museum, Regina, Saskatchewan, S4P 4W7 Canada; 6 Biology Department, University of Regina, Regina, Saskatchewan S4S 0A2 Canada; 7 Key Laboratory of Zoological Systematics and Evolution, Institute of Zoology, Chinese Academy of Sciences, Beijing, 100101 China

## Abstract

The mid-Cretaceous Burmese amber (~99 Ma, Myanmar), widely known for exquisite preservation of theropods, also yields microfossils, which can provide important contextual information on paleoenvironment and amber formation. We report the first Cretaceous ostracod in amber—the gigantic (12.9 mm) right valve of an exclusively marine group (Myodocopa: Myodocopida) preserved in Burmese amber. Ostracods are usually small (0.5–2 mm), with well-calcified carapaces that provide an excellent fossil record extending to at least the Ordovician (~485 million years ago), but they are rarely encountered in amber. The new specimen effectively doubles the age of the ostracod amber record, offering the first representative of the Myodocopa, a weakly calcified group with a poor fossil record. Its carapace morphology is atypical and likely plesiomorphic. The preserved valve appears to be either a moulted exuvium or a dead and disarticulated specimen, and subsequent resin flows contain forest floor inclusions with terrestrial arthropods, i.e., fragmentary remains of spiders, and insect frass. These features resolve an enigmatic taphonomic pathway, and support a marginal marine setting for resin production.

## Introduction

Ostracods are aquatic microcrustaceans, with a calcareous, bivalved shell (carapace) that can enclose the whole body and all appendages. Few Mesozoic to Recent taxa exceed 3 mm in size and these are termed ‘gigantic’ ostracods, such as species of the living marine planktonic genus *Gigantocypris* (subclass Myodocopa, up to around 30 mm), or of the non-marine genus *Megalocypris* (subclass Podocopa, 5–8 mm in size). Owing to their commonness, carapace calcification, and several moulting stages, ostracods have an excellent fossil record—among the best of any arthropod group. They are known from at least the Ordovician to Recent, and today ostracods inhabit virtually all aquatic environments at all depths. Modern habitats include both marine and non-marine waters, extending to include aquifers and hot springs: some species are even adapted to semi-terrestrial habitats, such as forest leaf litter or life between sediment grains.

Reports of ostracods from amber are scarce to date, and all are from the Cenozoic (Eocene and Miocene). Thus far, only a few non-marine ostracod specimens have been reported from amber with partial soft-part preservation. Weitschat and co-authors first reported ostracod specimens in amber, and documented the ostracods found in Baltic amber near Kaliningrad, Russia (Eocene, estimated 42–54 Ma) to the genus-level (*Cyclocypris*)^1–4^. Recently, a complete female specimen of a new species of a different genus, *Cypria kempfi*, was described from another locality containing middle or upper Eocene Baltic amber in northeastern Germany^5^. The findings from Baltic amber have all been freshwater taxa, and are thought to have been captured by the resin of conifer trees belonging to Pinaceae, Sciadopityaceae, or Araucariaceae, living in temperate to subtropical conditions with a strong aquatic influence^6^.

A diverse ostracod fauna has also been reported from multilayered Miocene amber near Chiapas, Mexico^7,8^. Material from this deposit includes more than 500 specimens^7^ dominated by species of the brackish water genus *Thalassocypria* and other taxa of the tribe Thalassocypridini, 262 specimens of which have very recently been analysed and described in detail^8^. These ostracods are associated with other aquatic crustaceans, such as amphipods, copepods, isopods, and tanaidids: detailed description and analysis of these assemblages are pending. Mexican amber is thought to have been produced by *Hymenaea* (Fabaceae), an angiosperm that was part of a mangrove forest in tropical dry conditions^9^. Beyond the few data on Eocene non-marine specimens from Baltic amber, and many Miocene specimens including brackish taxa, there are currently no data on marine, or brackish–marine ostracods, and none from the Mesozoic.

### Ostracods and aquatic organisms in amber

Resin is immiscible with water and hardens rapidly in air, so the capture of aquatic organisms in fossil tree resin has presented a puzzle in amber deposits^10,11^. The taphonomic pathway for inclusions of fully marine groups have been particularly difficult to explain. Previous records of aquatic animals in amber have been explained as the result of individuals being splashed up onto the resin of nearshore trees, or deposited there during flooding events (e.g., French Cretaceous amber^12^); isolated puddles of water drying out and the individuals having resin drop onto them, or being blown into the resin by winds (e.g., Baltic Eocene amber^2^); or even resin exuding near water-filled cavities within the trees, such as bromeliad microhabitats (e.g., Dominican Miocene amber^13^). Study of modern pine resins has indicated that the taphonomic pathway can be much less elaborate that these scenarios^14^. Trees adjacent to water have been observed releasing resin into the water, where it remains fluid enough to engulf both microscopic and macroscopic inclusions. This resin has some trapping bias towards larger and more motile arthropods, but it also has the ability to engulf water droplets with their own microscopic assemblages. The resin studied only began to polymerize strongly once exposed to air, leading to the suggestion that changing water levels played an important role in deposit formation for aquatic inclusions^14^. The effects of saltwater settings on polymerization remain to be examined in detail, as do the effects of variations in resin chemistry associated with the range of source plants to which amber deposits have been attributed.

### Burmese amber

Based on biostratigraphic evidence (ammonites and palynomorphs), the Cretaceous amber deposits of Myanmar (Burmese amber) have been assigned a late Albian–Cenomanian age (~105 to 95 Ma)^15^. U-Pb dating of zircons from the volcaniclastic matrix of the amber has provided a refined age estimate of approximately 98.8 ± 0.6 million years for the deposit^16^. This amber is thought to be the product of a conifer, perhaps belonging to the Cupressaceae or Araucariaceae, that lived in a moist tropical setting^17,18^. Burmese amber has become a focal point for fossil insect studies over the last twenty-five years^17–19^, providing important snapshots of evolution for the insect groups that dominate modern ecosystems. Over the last few years, this deposit has been mined on a scale that has made it an important new source for vertebrates with exceptional preservation^20–24^ and other taxa seldom preserved in amber. Here we expand the range of unusual taxa, by describing an enigmatic marine ostracod from the deposit.

## Results

### Specimen description

**Figure 1:**
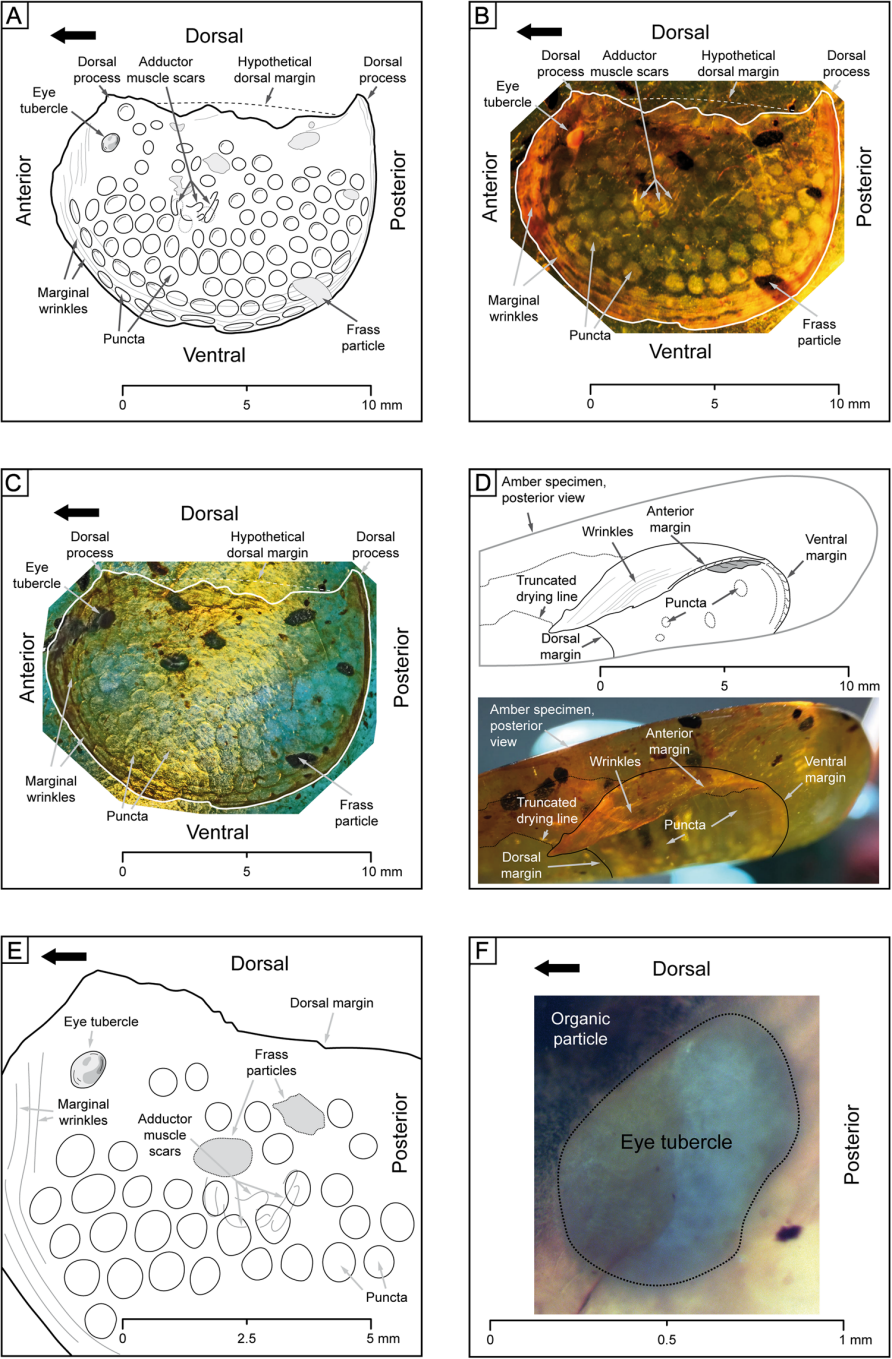
Detailed illustration of DIP-V-17118 ostracod. (**A**) Illustration of internal view of right valve of ostracod specimen in transmitted light as shown in (**B**,**C**). (**B**) Photomicrograph with transmitted light, with important morphological features highlighted. (**C**) Photomicrograph with UV light. (**D**) Ostracod anterior photomicrograph with transmitted light. Amber specimen viewed with posterior side of ostracod facing observer, with explanatory illustration. This view shows the strengthened posterior and ventral margins, the posterior marginal wrinkles and a prominent drying line within the amber (external to the carapace). (**E**) Detailed view of anterodorsal part. Magnified view of (**A**), highlighting irregular pattern of the puncta in ventral part of the valve (not all puncta in the dorsal part are clearly visible and drawn here), and the position of the lateral eye tubercle. (**F**) Detailed view of lateral eye tubercle. Magnified view of lateral eye tubercle in UV-light, with insect frass pellet organic particle overshadowing anterodorsal corner of eye tubercle.

**Figure 2:**
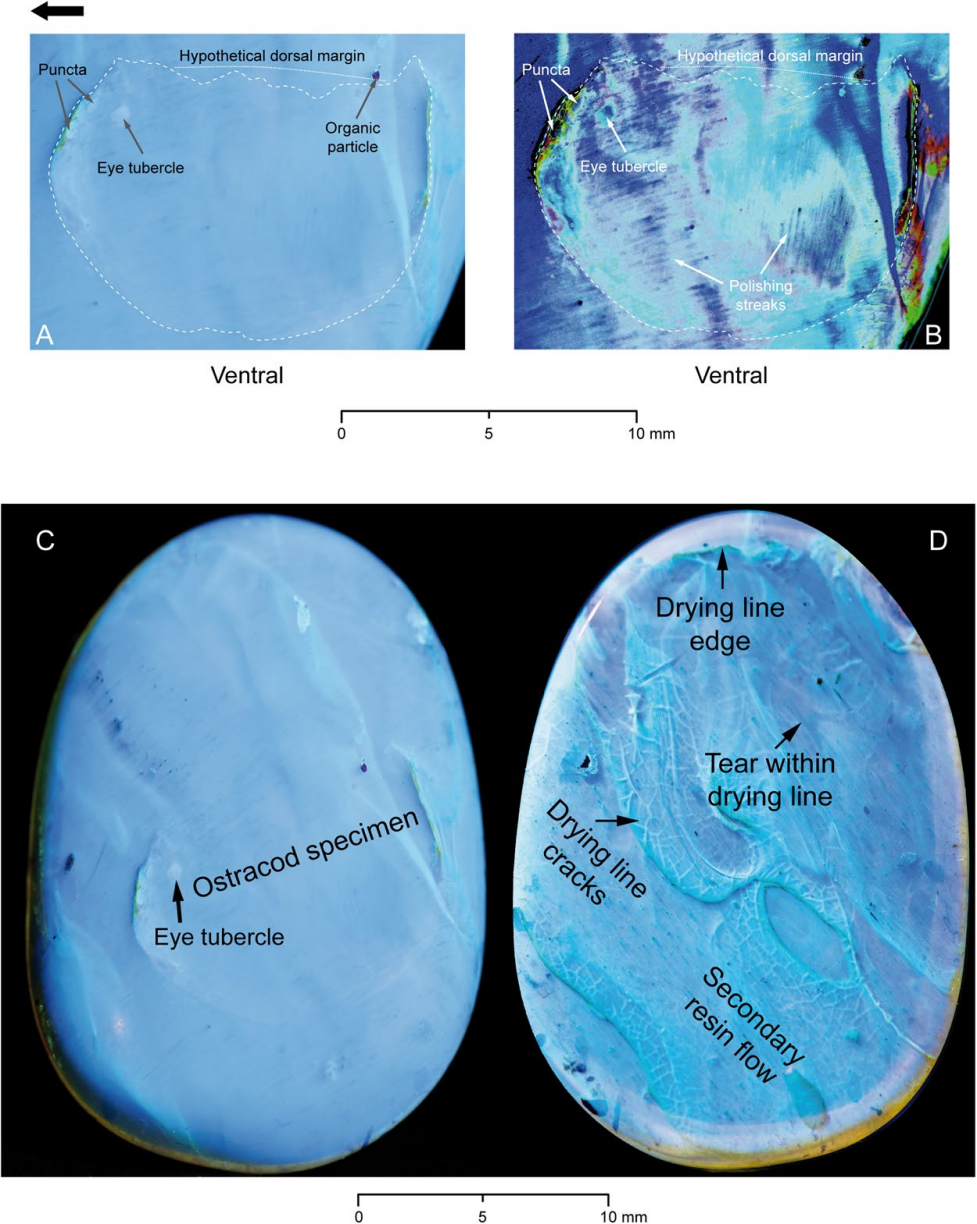
DIP-V-17118 ostracod UV-light examination. (**A**,**B**) Ostracod specimen under incident UV-light, internal view, anterior end to the left (black arrow). (**A**) Original UV-light photo, with compaction of anterior and posterior margins and anterolateral eye tubercle clearly visible. (**B**) Same photo with adjusted colour tonal range (Adobe Photoshop), curves adjusted for contrast enhancement and colour shade changes. Same morphological features highlighted on the left. Cutting and polishing streaks are clearly visible. (**C**,**D**) Whole amber specimen under incident UV-light, directly comparable to Fig. 3. (**C**) Internal surface and specimen view with visible ostracod specimen outline and eye tubercle; anterodorsal and posterior margins close to the surface. (**D**) External surface view of amber specimen with clearly visible secondary resin flow with tearings, drying cracks and drying lines indicated.

**Figure 3:**
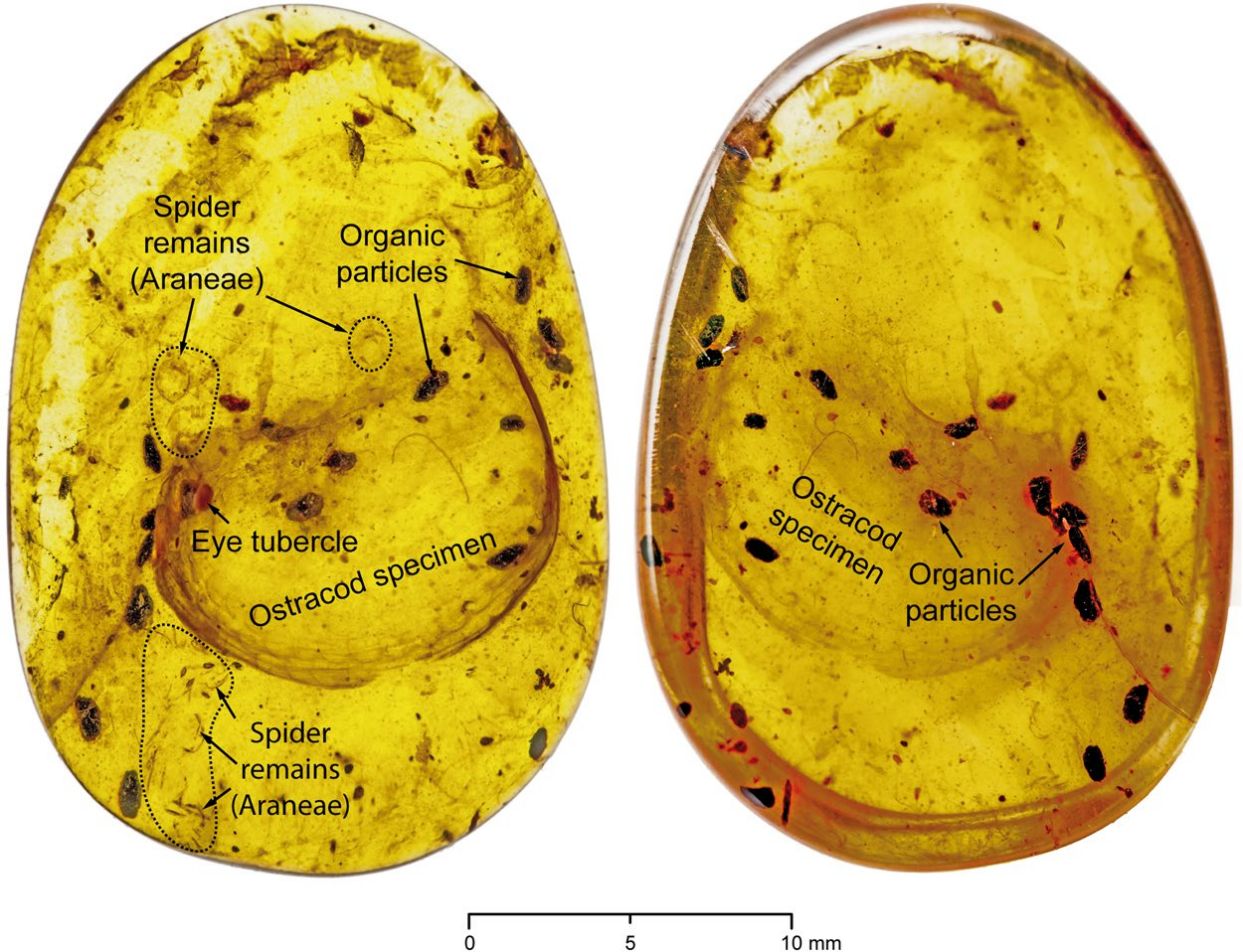
DIP-V-17118 amber overview for syninclusions and flow lines. Whole amber specimen in natural light, directly comparable to Fig. 2C,D. Left: Internal surface and specimen view with ostracod specimen clearly visible, with distinct orange convex anterolateral eye tubercle, brownish to dark grey frass particles (strongly distinct from the eye tubercle), as well as associated faunal inclusions (Spiders, Araneae, Oonopidae?) relatively near to the polished amber surface. Right: External surface view of valve, where ostracod is deeply buried under secondary resin flow with numerous insect frass pellets and organic particulates.

The ostracod specimen consists of a right valve (Fig. 1). The surrounding amber (specimen number DIP-V-17118) is an oblate piece, cut and polished to form a pendant by local artisans, measuring 28 × 19 × 7 mm with a weight of 2.23 g (Figs 2C–D, 3).

The ostracod valve inclusion is thin, and preserved in the same fashion as organics in this deposit, with a translucent appearance. The valve is bursiform in lateral outline (pouch-shaped, 12.90 mm maximum length, ~9.8 mm maximum height.), with broadly rounded anterior, posterior and ventral margins; however, the anterior margin looks somewhat narrower and more rounded than the posterior one. Anterior and posterior margins bear distinct processes, but the process is more distinct posteriorly (Fig. 1A–C). The dorsal margin appears to have been straight originally, but it has been strongly deformed and damaged by cutting and polishing of the surrounding amber.

Observation in transmitted light indicated most structural details for DIP-V-17118 (Fig. 1A–E). Anterodorsally, the valve exhibits a distinct anterolateral eye tubercle, which is visible as a strongly convex ovate spot anterior of the adductor muscle scar, homogenous yellowish-orange, and 0.85 mm in length and 0.68 mm in width. The tubercle differs clearly from the dark brown to black organic particles of insect frass (Fig. 1B), which have an irregular outline and pattern, and are situated adjacent to the specimen in a secondary resin flow. The valve surface is punctate (maybe reticulate), with sharply delimited, round depressions arranged in nearly concentric rows parallel to the valve’s ventral margin. In transmitted light, the puncta appear to vary in shape (circular to elliptical) and size (0.57 to 0.86 mm in diameter, with some elongated to 1.06 mm). Puncta generally follow the pattern of concentric rows, which are occasionally interrupted by preservational artefacts. Based on the puncta visible, the total number estimated is between 200 and 300. Particularly along the ventral margin, the puncta appear elongate in lateral view, which is partially structural, but also partially an optical effect due to their orientation. Puncta are present but almost indiscernible, in the dorsolateral area. The anterior, posterior and ventral marginal areas exhibit fine and dense wrinkles, almost parallel to these margins (Fig. 1B–D), particularly anteriorly (Fig. 1D) and ventrally. However, the deformation effect of the wrinkles is minor; the puncta are not warped, except for compressions along the more consolidated areas between the puncta parallel to the ventral margin. The outer selvage anteriorly, ventrally and dorsally appears strengthened through thickening, best visible under UV light (Fig. 1C). The dorsal margin is damaged and strongly deflected ventrally within the amber, lacking clear delimitation. In transmitted light, a round area of about 1.5–2.0 mm diameter is visible anterocentrally in the internal part of the valve; in this region, elongated, arcuate spots of about 0.2 mm width and up to 1.3 mm in length demarcate muscle scars arranged in a subparallel, concentric pattern.

In contrast to transmitted light, observation with incident ultraviolet (UV) light (Fig. 1C) suggests a more regular, almost reticulate ornamentation pattern (with slightly polygonal puncta). Overall, the puncta appear larger, with smaller areas between them, and arranged in regular concentric rows. However, the puncta still vary in shape and size (up to 20%). The organic particles of insect frass appear dark-greyish, as does the anterolateral eye tubercle, while the muscle scars are not visible under UV-light.

**Figure 4:**
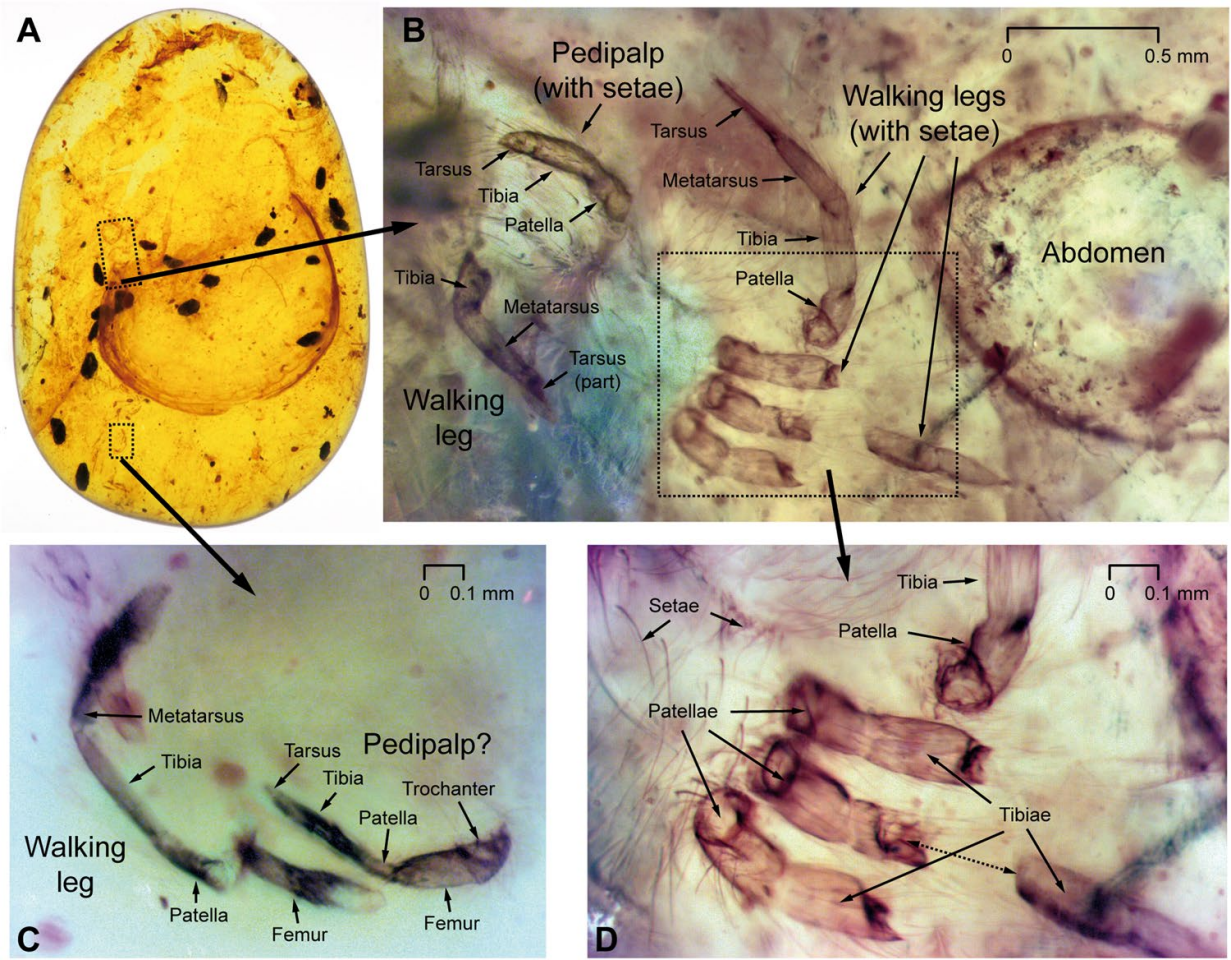
Arthropod syninclusions of the DIP-V-17118 ostracod. Detailed views of partial spider (Araneae, Araneomorphae) inclusions associated with the ostracod specimen. (**A**) Amber specimen focusing on internal view of ostracod, under incident light, for reference. (**B**) Detail of small Araneae remains (?Oonopidae = goblin spiders) under UV-light, rotated 100° clockwise; overall length of specimen about 2–3 mm; specimen may be an exuvium as the body parts are disarticulated and the cephalothoracal (prosoma) region is barely identifiable. Left: One pedipalp with setae, tarsus short, tibia, and patella and distal parts of a walking leg. Center: Disarticulated distal parts of the walking legs. Right: Abdomen (opisthosoma). (**C**) Magnified view of small spider appendages from (**A**), rotated 90° clockwise, under UV-light, walking leg and pedipalp? (**D**) Magnified version of (**B**), under UV-light; disarticulated right walking legs showing patellae, parts of tibiae, and many attached and loose setae.

Disarticulated remains of small spiders (Araneae), and potentially of mites (Acariformes? or Opilioacariformes?), are included anteroventrally, anterodorsally, and posterodorsally of the ostracod specimen (Figs 3 and 4). Anterodorsally (Fig. 4A–B,D), the remains of a more complete small spider of about 2–3 mm size may suggest that many of these minute inclusions belong to Oonopidae (goblin spiders). However, these indeterminate inclusions are strongly disarticulated, consisting of barely recognizable cephalothorax (prosoma) and abdomen (opisthosoma) regions, and several disarticulated distal parts of walking legs.

## Discussion

### Identification

Characters that support the ostracod valve identification include: (1) the overall outline, with rounded anterior, ventral and posterior margins and the more or less straight (or perhaps convex) dorsal margin, which is characteristic of some ostracods (mainly ancient or ‘primordial-Paleozoic’ type Palaeocopida, but also of some Ordovician to Recent Myodocopa, for example), and the margins are structurally strengthened; (2) the specimen is bivalved, although only one valve was available for study; (3) the valve exhibits a relatively regular ornament of puncta arranged concentrically and subparallel to the margins; (4) a distinct anterior dorsolateral protruding, convex structure is present, which we interpret as an anterolateral eye tubercle – a characteristic feature of certain ostracod taxa; and (5) the anterocentral field contains elongate, arcuate spots we interpret as potential adductor muscle scars. Other bivalved taxa, and morphologically similar components of other organisms can be excluded with confidence, based on features discussed in the Supplementary Online Material.

The main sources for uncertainty in the ostracod identification are the preservational state of the specimen, the lack of knowledge concerning its other valve or appendages, and the lack of density contrast necessary to conduct X-ray micro-CT analysis. Damage to the dorsal margin has removed or deformed informative features including the hinge, an important diagnostic feature in ostracods or other candidate organisms (e.g., Phyllopoda, especially Spinicaudata). The presumed muscle scar field is barely visible in internal view, and the shape of the individual scars is vague. Due to a thin carapace, the valve margins are somewhat wrinkled. We could identify neither marginal structures typical for ostracods (such as the marginal infold/duplicature), nor marginal or lateral pore canals (valve penetrations bearing setae that are usually a few micrometers in diameter), likely as a result of resin infilling these features. Finally, the size of the specimen strongly exceeds that of most known ostracods of the major Mesozoic to Recent subclasses of Ostracoda, the Myodocopa and the Podocopa. Valve size coupled with the distinct anterodorsal eye tubercle, the characteristic ornament, and limited knowledge of the muscle scar and dorsal margin (which could be straight or convex, a dorsal connection, or a true hinge as preserved) make it somewhat difficult to assign DIP-V-17118 to known fossil or Recent taxa. Altogether, our specimen has the closest potential affinities to species of the ostracod subclass Myodocopa.

DIP-V-17118 most likely belongs to the subclass Myodocopa, order Myodocopida, based on specimen size, preservation, general shape and the existence of an anterolateral eye tubercle. The almost exclusively marine Myodocopa (Ordovician–Recent), include benthic, nektobenthic and fully pelagic forms, that occur worldwide. Members have a wide bathymetric range, from shallow to abyssal depths—some groups (Sarsielloidea and Cylindroleberidoidea) are more prevalent in shallow coastal or intertidal zones—and they are among the most abundant macroinvertebrates on some continental shelves^25,26^. Most Recent myodocopes are nektobenthic scavengers and predators. Myodocopa carapaces can be of variable lateral outline, ranging from elongate-ovoid to circular or subquadrate; they are usually thin and weakly calcified (translucent); their ornamentation ranges from smooth to prominent, with ribs, puncta or reticulation^27^. Due to these features, the fossil record of Myodocopa is generally poor, with the exception of the Silurian Herefordshire, UK Konservat-Lagerstätte (where soft part preservation occurs due to calcitic void infill of carbonate concretions)^28–31^, and other exceptionally preserved examples from the Triassic^32^ and Jurassic^33^. Representatives of the Order Myodocopida can reach sizes of up to 32 mm today^27^, but these are active pelagic swimmers. Many Paleozoic Myodocopa are ‘gigantic’ by ostracod standards (e.g. *Colymbosathon ecplecticos*^28^, which is 5.2 mm; see also^31^), but, with some exceptions, these are often less than 15 mm in size, while most Recent myodocopes are around 1–3 mm long^27^. Many, but not all, modern Myodocopa possess an anterior rostrum and incisure (notch); these features are particularly common in good swimmers, which possess a dorsal margin that is convex or sometimes straight. Myodocopa are the only ostracods with a pair of lateral compound eyes; however, in Recent species, the eyes are located below the translucent carapace, which does not express lateral eye tubercles.

Known variation within Myodocopa suggests that DIP-V-17118 can be accommodated within the subclass, but the range of carapace features, combined with examples of morphological stasis, restrict our inferences at lower taxonomic levels. Other cases of exceptional carapace preservation with appendages^29^ unequivocally demonstrated that the carapaces of some fossil Myodocopida can exhibit straight dorsal margins in combination with an adductorial sulcus, and be bursiform (e.g. *Nymphatelina*^30^), which are features otherwise known from many Palaeocopida (Ostracoda: Podocopa?). Unlike many Myodocopa, which possess a rostrum and rostral incisure, including unequivocal Cylindroleberididae of the Triassic, Jurassic, and Silurian^31–33^ (e.g., *Nasunaris*^30^); several other unequivocal Silurian Myodocopa (e.g., species of *Nymphatelina*^29^) or Cylindroleberididae (e.g., species of *Colymbosathon*^28^ or *Pauline*^31^) lack a rostrum or rostral incisure. Exceptionally preserved Silurian Myodocopes of the Herefordshire Konservat-Lagerstätte have led to the conclusion that carapace morphology in fossil Myodocopa can be diverse, corroborating ‘that carapace morphology alone is an inadequate basis for suprageneric assignment of (Recent) myodocopes’^31^. Considering that the soft parts of living Cylindoleberididae are nearly identical to those of the Silurian species *Columbosathon ecplecticos*^28^, we too advise caution regarding the designation and interpretation of specimens preserved without soft parts, such as DIP-V-17118. Until more complete specimens become available, designation below the order-level (Myodocopida) must remain tentative for DIP-V-17118. There is insufficient support for naming a new species and genus at this time, as it is even impossible to attribute the specimen to a superfamily thus far. Therefore, we designate our specimen as Myodocopida indet.

The muscle scar shape (long elongate, arcuate) and arrangement visible in DIP-V-17118 show affinities to the myodocopid superfamily Cypridinoidea. Considering particularly the dorsal process of the posterior margin overtopping the dorsal margin, our specimen exhibits some similarities to the Recent cypridinid genus *Heterodesmus* (as re-diagnosed^34^). Examples of shared characters with *Heterodesmus* include the bursiform overall shape and the diagnostic antero- and posterodorsal processes. However, DIP-V-17118 lacks a rostrum and rostral incisure—which are diagnostic characters in the Cypridinoidea, where the incisure is usually very deep and curved, forming an inlet (these features allow for protrusion of swimming antennae while the carapace is closed). Moreover, DIP-V-17118 exhibits a coarsely punctate ornamentation, which is not present in many Cypridinidae; and it has a distinctly anterior, anterodorsal eye tubercle, which is located farther anterior than the lateral compound eyes of most Recent Cypridinoidea– including *Heterodesmus*, though *Heterodesmus adamsii* comes close. The balance of evidence does not provide strong support for assignment of our new specimen to the Cypridinoidea, and we consider this placement unlikely.

At first view, DIP-V-17118 shows some similarities to the living relict ostracod species *Manawa staceyi* Swanson^35,36^ (Palaeocopida: Kirkbyocopina: Puncioidea) in lateral carapace outline and overall valve shape. This particularly applies to the dorsal processes present on the right valve, and the punctate valve surface. However, *M*. *staceyi* is moderately inaequivalve, with dorsal processes restricted to the right valve, and the lack of a preserved left valve in DIP-V-17118 precludes a more complete comparison. Conversely, the visible portions of the central muscle scar preserved in DIP-V-17118 differ strongly from the muscle scar of *M*. *staceyi* (which is paw-shaped, consisting of six elliptical scars, one central and five arranged dorsally in a semicircle). Moreover, DIP-V-17118 is about 25 times larger than specimens of *M*. *staceyi* (around 500 µm in size); its puncta are rather large in relation to the carapace size (measuring ~0.05–0.06 versus <0.02 of carapace length); DIP-V-17118 also does not exhibit distinct lateral pore canals and the camerate anterior, ventral and posterior margins present in *M*. *staceyi*. Altogether, there are strong morphological differences that make allocation of DIP-V-17118 to the evolutionary lineage that led to *M*. *staceyi* rather unlikely, but missing features, such as those on the left valve, preclude a more comprehensive evaluation.

### Paleoenvironment

Full paleoenvironmental details for the numerous amber-producing sites in the Kachin state have yet to be reported. However, a general understanding of the geology in the region during the time of amber deposit formation has been gained from the Noje Bum site studied by Cruikshank and Ko^15^. Here, amber is found in laminated coals interspersed among fine clastic rocks thought to be the product of subtidal deposition in a bay, lagoon, or estuary. Support for a strong marine influence comes from discoveries of ammonites, bivalves, forams, algal remains, and marine dinoflagellates from the surrounding rocks^15^. The amber from the Hukawng Valley contains a range of semi-aquatic insects, such as Ochteridae (Hemiptera) and Heteroceridae (Coleoptera). It also contains aquatic insects such as Chresmododea and Gerridae (Hemiptera); Dytiscidae and Gyrinidae (Coleoptera); Odonata; and larvae of Psephenidae, Trichoptera, and Ephemeroptera^37^. Taxa such as Trichoptera have larvae that favor clean, flowing water with high oxygen content, indicating that there were streams in the ancient Burmese forest. Other insect groups with ecologically restricted larval habitats indicate that parts of the forest were producing resin in marginal marine settings as well (e.g., Diptera: Certatopogonidae: *Leptoconops*^38^). Marine bivalves (Myoida: Pholadidae) are known to have bored into amber pieces from the Noije Bum summit site^39^. Pholadids can be found in the Angbamo site as well (and also in the Xipiugong site, 10 km to the northeast). However, pholadids are absent in the Hkamti site (L. Xing, pers. obs.), perhaps indicating a more inland position than the Angbamo and Xipiugong sites.

### Taphonomy

The amber that constitutes DIP-V-17118 was produced under two distinct sets of conditions. The resin flow that contains the ostracod is relatively clear, and is separated from a secondary resin flow that contains multiple, dark, organic particles of insect frass, as well as the fragmentary remains of spiders that may belong to the family Oonopidae (Fig. 4; minute, wandering spiders that are particularly common in tropical rain forests^17^ and present in Burmese amber^40^. Between these two flows there is a prominent drying line (Figs 1D, 2C,D), which has been distorted due to flow around the ostracod valve while the resin was still pliable. Tears and cracks within the dark surface of the drying line indicate that the resin had oxidized and partially polymerized^41^ before a secondary flow containing insect frass and a large amount of fine particulate matter was added (Figs 2C,D, 3). This suggests a scenario in which resin was released underwater or at the water’s edge, encapsulating the ostracod, then the resin mass dried subaerially for a significant length of time before a subsequent resin flow captured a range of inclusions more commonly associated with a forest floor habitat^42^. The combination of marine and terrestrial resin flows may have been brought about by variation in water levels, a mechanism proposed in the study of modern marine organisms preserved in resin^14^, and invoked for other Cretaceous ambers with marine contents^43^.

Weak calcification is characteristic of the Order Myodocopida, but the valve preserved in DIP-V-17118 is markedly thin and flexible. These features may provide some clue as to how the carapace entered the resin. Features such as punctae along the ventral margin of the valve appear elongate due to marginal deformation, and there is significant damage to the dorsal margin of the valve. Thinning of the carapace may be the result of dissolution: reactions with pore waters have been invoked for dissolved and infilled inclusions in this deposit^17,22^. However, there are no voids or mineral deposits in the amber piece that would point toward dissolution in DIP-V-17118. Decalcification, coupled with a lack of preserved soft parts, and substantial separation between the two valves, suggests that the preserved valve is either a cast exuvium or the valve of a dead and disarticulated specimen. In the former case, the thin carapace would result from reabsorption prior to moulting, and it is unlikely that the carapace was driven into the resin mass by a motile organism, as has been observed in modern resin study^14^.

## Methods

The amber specimen, DIP-V-17118, comes from the Hkamti site, Hkamti District, Sagaing Region, Myanmar. The Hkamti site is about 80 km southwest of the Angbamo site. The amber specimen measures approximately 28 × 19 × 7 mm and weights 2.23 g. It is property of, and housed at, the Dexu Institute of Palaeontology (DIP), Chaozhou, China. The specimen was collected by Amber House Co., Ltd., Myitkyina, Myanmar. Technicians there cut larger amber specimens into small pieces and polish them for sale. Our specimen originally consisted of two valves, but these were separated in the center and unfortunately, the other piece has been sold. DIP-V-17118 represents the right valve of a large ostracod, and it is closest to the polished amber surface along the anterior and posterior margins of its inner surface (Figs 2A,B, 3).

The specimen was examined with a Leica MZ 12.5 dissecting microscope equipped with a drawing tube attachment. Photographs were taken using a Canon digital camera (5D Mark III, MP-E 65MM F/2.8 1–5×) fitted to a macro rail (Cognisys), and were processed using Helicon Focus 5.1 and Adobe Photoshop CS5 software to increase depth of field in the images. Illustrations were prepared in all observable anatomical orientations, and a large black arrow is used as a standard Figure annotation, pointing anteriorly whenever relevant.

Micro-CT analysis has been tested and conducted by at the Institute of Zoology, Chinese Academy of Sciences (IOZ, CAS), Beijing, China, but without good results. This problem results from the fact that the specimen is preserved as a very thin organic material without significant inorganic mineralization, and with resin permeating all voids in the sample. The specimen density contrast compared to the surrounding amber is insignificant and not resolvable with a Micro-CT.

## Electronic supplementary material

Supplementary Information
