# Supplementary material for: A gigantic marine ostracod (Crustacea: Myodocopa) trapped in mid-Cretaceous Burmese amber
Source: Sci Rep. 2018 Jan 22;8:1365. doi: 10.1038/s41598-018-19877-y (PMC5778021; doi:10.1038/s41598-018-19877-y)
Supplement: Supplementary file 1 — Supplementary Information [file 41598_2018_19877_MOESM1_ESM.rtf]

Supplementary Discussion

A gigantic marine ostracod (Crustacea: Myodocopa) trapped in mid-Cretaceous Burmese amber

Lida Xing 1, 2 *, Benjamin Sames 3, 4 *, Ryan C. McKellar 5, 6, Dangpeng Xi 1, 2, Ming Bai 7, Xiaoqiao Wan 1, 2

1. State Key Laboratory of Biogeology and Environmental Geology, China University of Geosciences, Beijing 100083, China
2. School of the Earth Sciences and Resources, China University of Geosciences, Beijing 100083, China
3. Department of Geodynamics and Sedimentology, University of Vienna, Geozentrum, Althanstrasse 14, 1090 Vienna, Austria
4. Sam Noble Museum, 2401 Chautauqua Avenue, Norman, OK 73072, USA
5. Royal Saskatchewan Museum, Regina, Saskatchewan S4P 4W7, Canada
6. Biology Department, University of Regina, Regina, Saskatchewan, S4S 0A2, Canada.
7. Key Laboratory of Zoological Systematics and Evolution, Institute of Zoology, Chinese Academy of Sciences, Box 92, Beichen West Road, Chaoyang District, Beijing, 100101, China

Correspondence and requests for materials should be addressed to B.S. (benjamin.sames@univie.ac.at) or to L.X. (xinglida@gmail.com).

Commentary on support for ostracod identification

Spinicaudata
DIP-V-17118 cannot be the 'valve' of a carapace of the Spinicaudata (Crustacea: Branchipoda: Phyllopoda: Diplostraca—a group previously assigned to the 'Conchostraca' or clam shrimps). This is a group that today lives exclusively in non-marine waters. Though they are known since at least the Devonian, their organic carapace is bilobate (i.e., not truly bivalved, without a hinge), with adductor muscle scars, and not mineralized with calcite. They are similar in size to our specimen, but we can exclude spinicaudatans because their carapace is different in outline, exhibits distinct 'growth bands' (also 'growth rings or lines') and an umbo (like in true Bivalvia), and they exhibit neither lateral eye tubercles, nor ornamentation of large puncta measuring more than 0.5 mm in diameter (the spinicaudatan ornamentation important for differentiation of fossil taxa is very delicate in the scale of around 5–20 µm).

Laevicaudata
The closely related Laevicaudata (Crustacea, Branchiopoda, Phyllopoda) can be excluded as well: they have an organic carapace separated into two valves with a hinge, and reach sizes up to several millimeters, but their carapace is smooth (common name 'smooth clam shrimp') and globular, without a lateral eye tubercle.

Limulid element
A structure that somewhat resembles our specimen in shape, size (up to around 1 cm in large species), and surface structure (punctate), would be the lateral compound eye of a member of Limulidae (horseshoe crab, Chelicerata, Xiphosura). Though superficial similarities are striking, the consideration that our specimen could be a part of a limulid exuviae (compound eye cuticle) is rejected for several reasons: First, the outline of the limulid compound eye is more elongate and asymmetric; second, these eyes consist of approximately one thousand individual ommatidia that are around 0.3 mm in size and regularly arranged, as is typical for compound eyes; third, there would be no extra eye tubercle; and finally, an eye cuticle specimen, if isolated from the cephalothorax (prosoma), would not have its counterpart preserved directly adjacent to it as in DIP-V-17118.

Insect element
Hexapod eye exuviae can be excluded for many of the same reasons as the limulid element. Some eyes appear similar to our specimen, in terms of size or surface pattern, and would be paired elements; however, ommatidia appear convex at the cuticle surface, instead of concave as in the puncta of DIP-V-17118, and they are several orders of magnitudes smaller than our specimen's puncta. The same holds true for the whole ostracod specimen which with 12.9 mm is much larger than than any insect eye (few millimeter-scale or smaller).
